# The Impact of the COVID-19 Pandemic on People With Epilepsy. An Italian Survey and a Global Perspective

**DOI:** 10.3389/fneur.2020.613719

**Published:** 2020-12-18

**Authors:** Barbara Mostacci, Laura Licchetta, Carlotta Cacciavillani, Lidia Di Vito, Lorenzo Ferri, Veronica Menghi, Carlotta Stipa, Patrizia Avoni, Federica Provini, Lorenzo Muccioli, Luca Vignatelli, Stefania Mazzoni, Paolo Tinuper, Francesca Bisulli

**Affiliations:** ^1^IRCCS Istituto delle Scienze Neurologiche di Bologna, Full Member of the ERN EpiCare, Bologna, Italy; ^2^Department of Biomedical and Neuromotor Sciences, University of Bologna, Bologna, Italy

**Keywords:** COVID-19, emergency, epilepsy, survey, telemedicine

## Abstract

**Objectives:** We explored the impact of the coronavirus disease-19 (COVID-19) emergency on the health of people with epilepsy (PwE). We also investigated their attitude toward telemedicine.

**Methods:** The PubMed database up to September 10, 2020 was searched for questionnaire-based studies conducted in PwE during the COVID-19 emergency, and the literature retrieved was reviewed. In addition, all patients who had a telephone consultation with our center between May 7 and July 31, 2020 were invited to fill in a 57-item online questionnaire focusing on epilepsy and comorbidities, any changes in lifestyle or clinical conditions and any emergency-related problems arising during the COVID-19 emergency, and their views on telemedicine. Associations between variables were detected through *X*^2^ test and Fisher's exact test. Univariate and multivariate logistic regression models were used to evaluate the effects of different factors on clinical conditions.

**Results:** Twelve studies met the literature search criteria. They showed that the rate of seizure worsening during the emergency ranged from 4 to 35% and was mainly correlated with epilepsy severity, sleep disturbances and COVID-19-related issues. Our questionnaire was filled in by 222 PwE or caregivers. One hundred (76.6%) reported unchanged clinical conditions, 25 (11.3%) an improvement, and 27 (12%) a deterioration. Reported clinical worsening was associated with a psychiatric condition and/or medication (OR = 12.59, *p* < 0.001), sleep disorders (OR = 8.41, *p* = 0.001), limited access to healthcare (OR = 4.71, *p* = 0.016), and experiencing seizures during the emergency (OR = 4.51, *p* = 0.007). Telemedicine was considered acceptable by 116 subjects (52.3%).

**Conclusions:** Most PwE did not experience a significant change in their clinical conditions during the COVID-19 emergency. However, severity of epilepsy, concomitant disability, comorbid psychiatric conditions, sleep disorders and limited access to healthcare may affect their health.

## Introduction

The first half of 2020 saw a rapid spread of severe acute respiratory syndrome coronavirus 2 (SARS-CoV-2) infections worldwide. The outbreak of coronavirus disease 2019 (COVID-19), the disease caused by SARS-CoV-2, quickly reached pandemic proportions, seriously impacting the health systems of many countries. Italy was the first country in the Western world to be hard hit by the disease. On February 21, 2020, the Italian government issued the first of a series of legislative decrees that introduced increasingly stringent measures, closing down non-essential activities and severely restricting travel. These measures were rapidly extended to the whole of Italy. By March 11, the country's entire population was required to comply with strict home confinement (lockdown) measures.

The emergency posed an unprecedented challenge to our healthcare system (HS). The rapid HS re-organization together with the lockdown measures produced a restriction of care provision to all non-urgent conditions, including chronic neurological diseases. It has been reported that seizures in people with epilepsy (PwE) might be triggered by stress (1), including major environmental stressors (2). Sleep and other lifestyle changes may also influence seizure occurrence. Stress linked to health concerns, restricted healthcare access, and lifestyle changes due to home confinement and remote working might all be factors influencing seizure occurrence and the overall well-being of PwE. We set out to explore the impact of the recent lockdown measures on the health of PwE. A further aim was to explore how PwE felt about telemedicine, as the present, unprecedented situation has given us (and others worldwide) our first experience of application of this modality in epilepsy care. In this paper, after reviewing the literature on the use of questionnaires in the fields of epilepsy and COVID-19, we present the original results of an online survey conducted in Italy.

## Methods

### Review of the Literature

The PubMed database up to September 10, 2020 was searched for English-language questionnaire-based studies conducted in PwE during the COVID-19 emergency. The search was conducted using the terms: (“Epilepsy”[Mesh]) AND “COVID-19” [Supplementary Concept]) AND “Surveys and Questionnaires”[Mesh]; (“Epilepsy”[Mesh]) AND “severe acute respiratory syndrome coronavirus 2” [Supplementary Concept]; “Epilepsy”[Mesh]) AND “Coronavirus Infections”[Mesh].

Only studies based on questionnaires aimed at patients and/or caregivers were considered.

### Questionnaire

#### Setting

In response to the COVID-19 emergency, our institute, like many other healthcare facilities, underwent a major reorganization: by March 14, the inpatient facilities had been converted into a COVID-19 hospital and the operating theaters into a COVID-19 intensive care unit. Therefore, inpatient admissions for epilepsy diagnosis, monitoring and surgery were suspended. The activity of the outpatient clinic was also reorganized, in compliance with orders from local authorities aimed at limiting interpersonal contact. With the exception of selected urgent cases, first visits were suspended, as were follow-up EEGs, while follow-up consultations were performed almost exclusively through telephone calls with the treating clinician. From June 3, the full lockdown measures in Italy were partially relaxed. Travel between different regions was possible once again and facilities gradually reopened. Our inpatient clinic reopened on June 1. First visits and face-to-face check-ups for patients with vagal nerve stimulation implants or those in need of a neurological examination (e.g., for alleged side effects) were restored; however, at the time writing, most follow-up appointments are still conducted by telephone, as the authorities recommend use of this modality whenever feasible.

#### Questionnaire Design

Drawing on our Epilepsy Center clinicians' experiences of remote contact with patients during the first 2 months of lockdown, we created, using Google Forms, a 57-item, Italian language, self-administered questionnaire aimed at PwE. The instrument was designed to collect the following information: compiler identity (patient/caregiver/guardian), date of compilation, personal information (8 closed-ended +1 open ended questions), living situation (4+2 questions), possible COVID-19 infection (3+2 questions), changes in clinical conditions during the COVID-19 emergency (2 closed-ended questions), clinical information (5+3 questions), changes in lifestyle, and any problems or concerns related to the home confinement and limited access to healthcare resources since the implementation of the first emergency legislative decree on 23 February (10+10 questions), Finally, the responder was asked to express an opinion on the replacement of face-to-face appointments with telephone consultations.

#### Patient Recruitment

All patients who had had a telephone consultation with our center in the period from May 7 to July 31 2020 were sent a link to the questionnaire and invited to participate in the survey. It was underlined that participation was voluntary and anonymous. Although the questionnaire was available on the internet, it was not promoted in any other way. In order to avoid duplicates, patients were required to register with an e-mail address instead of a password. At the start of the questionnaire, patients were required to consent to the use of their data, in aggregate form, for research and scientific publication purposes. Only those responding “I agree” were able to access further questions. Data were processed according to the European regulation n. 2016/679 (GDPR). Patient recruitment closed on 31 July, 2020.

#### Statistical Analysis

Data manipulation and statistical analysis were performed using STATA software (version 14.0). To facilitate interpretation of the data, some numeric variables (e.g., age) were recoded as categorical, while some categorical variables (e.g., change in clinical conditions) were re-coded into fewer categories. Moreover, some variables were created specifically in order to consider additional aspects (i.e., time since last seizure, subsequently coded into categories). A “reported psychiatric condition and/or medication” variable was also generated by grouping patients who, in the open-ended questions, reported a current psychiatric diagnosis or took psychotropic drugs. Variables on therapy and sleep changes, derived from open-ended questions, were coded into categories. A further variable derived from the open-ended questions concerned the presence of sleep disorders. Descriptive statistics were run on all variables, except the uncoded open-ended questions. Subsequently, associations between qualitative variables were investigated through bivariate analyses using a *X*^2^ test, or Fisher's exact test in the case of low expected frequencies. A multiple correspondence analysis was also applied to detect associations between a subset of variables through a multidimensional technique. Lastly, both univariate and multivariate logistic regression models, the latter with a backward elimination criterion, were implemented to evaluate the effect of different factors on variation of clinical conditions, in terms of worsening vs. not worsening.

## Results

### Review of the Literature

We found 12 studies based on questionnaires aimed at PwE and/or their caregivers, conducted during the COVID-19 emergency. Table 1 reports their main characteristics (methods and population).

**Table 1:** Characteristics of the questionnaires.

**First author**	**Country**	**Method of distribution/administration**	**Period of recruitment**	***N* of items**	**Single/ multicenter**	**Topics covered**	***N*. of patients**	Age in years^*^
Áledo-Serrano A.	Spain	Online questionnaire	7 April−11 April 2020	na	na (patients advocacy groups)	General data, seizure frequency, COVID-19, lockdown related problems	277 DEE 161 (58.1%) F 116 (41.9%) M	12.4 (mean)
Alkhotani A.	Saudi Arabia	Electronic self-administered questionnaire distributed by treating neurologist	April 2020	na	Multicenter	General data, seizure frequency, lockdown related problems	156 97 (62.2%) F 59 (37.8%) M	<20–>60
Fonseca E.	Spain	Telephone survey administered directly to patients (or caregiver) by the neurologist	16 March−17 April 2020	19	Single-center	General data, seizure frequency, COVID-19, lockdown related problems satisfaction with telemedicine	255 121 (47.5%) F 134 (52.5%) M	17–94
Asadi-Pooya Ali A.	Iran	By telephone	27 March−31 March 2020	semi-structured	Single-center study	General data, seizure frequency, COVID-19, problems obtaining drugs	100 47 (47.0%) F 53 (53.0%) M	11–75
Assenza G.	Italy	Online questionnaire	11 April−16 April 2020	48	na (online survey)	General data, seizure frequency, COVID-19, lockdown related problems	928 456 PwE 472 PwoE 691 (74.5%) F 237 (25.5%) M	18–89 (overall)
Cabona C.	Italy	Telephone questionnaire administered by the neurologist	9 March−30 April 2020	semi-structured	Multicenter	General data, seizure frequency, COVID-19, lockdown related problems	189 103 (54.5%) F 86 (45.5%) M	45 (median)
Hernando-Requejo V.	Spain	By telephone	20 March−13 April 2020	na	na	General data, seizure frequency, COVID-19	49 23 (46.9%) F 26 (53.1%) M	na
Huang S.	China	Online questionnaire	23 February−5 March 2020	88	Single-center	General data, seizure frequency, COVID-19, lockdown related problems	362 166 (45.9%) F 196 (54.1%) M	10–19–≥60
Miller W. R.	USA	Online questionnaire	27 March−30 March 2020	65	Single-center	General data, seizure frequency, COVID-19, lockdown related problems	94 47 (50.0%) F 47 (50.0%) M	19–88
van Hees S.	Multinational	Online questionnaire	10 April−18 May 2020	na	Multicenter	General data, seizure frequency, COVID-19, lockdown related problems	399 320 (80.2%) F 79 (19.8%) M	38.22 (mean)
von Wrede R.	Germany	Audit directly administered to patients (or their caregiver)	23 March−8 May 2020	na	Single-center	General data, seizure frequency, satisfaction with telemedicine	239 126 (52.7%) F 113 (47.3%) M	18–93
Hao X.	China	Online questionnaire (medical information was collected from electronic medical records)	1 February−29 February 2020	na	Single-center	General data, seizure frequency, COVID-19, lockdown related problems	252 Pwe 252 PwoE For both Pwe and PwoE: 126 (52.4%) F 120 (47.6%) M	29.3 (mean of PwE) 29.4 (mean of PwoE)

* *range, unless otherwise specified, PwE = people with epilepsy, PwoE = people without epilepsy, DEE = patients with developmental and epileptic encephalopathies, na = not available/not applicable*.

#### Seizure Course During the Emergency

Nine questionnaires investigated seizure course during vs. prior to the emergency. The rate of seizure worsening ranged from 4 to 35% (3–11).

Worsening was significantly associated with several seizure and epilepsy factors: drug-resistant epilepsy (3, 7) number of anti-seizure medications (ASMs) (3, 9), and not being seizure free (9) or having more seizures at baseline (3, 7). In single studies, tonic-clonic seizures during the COVID-19 pandemic (9) and tumor-related etiology (3) were associated with worsening.

Seizure worsening was also associated with more disturbed sleep (3, 9) and with depression and anxiety factors: history of depression, anti-depressant use, more severe depression and anxiety symptoms (9). Fear of epilepsy was associated with worsening in two studies (3, 7).

Several authors reported an association with COVID-19 emergency-related issues (9), including reduced income (3) and difficulties obtaining ASMs (10).

Two studies reported a correlation with higher age (7, 10), however, since the first concerned a pediatric population, the data are not comparable. A Chinese study reported associations with Wuhan provenance and a history of exposure to COVID-19 (7).

Seizure improvement was also reported, albeit in a minority of studies, with rates ranging from 4 to 14.1% (6, 10, 11) and it was associated with improved sleep (6), less severe anxiety symptoms (6), and taking less than two ASMs (11).

#### Depression and Anxiety

Depression and anxiety were considered and measured, in different ways, in six studies. Alkhotani et al. reported “psychiatric disorders” in 40% of their subjects with epilepsy. In a survey of people with and without epilepsy (9), depression was reported in 19% of PwE vs. 17% of controls; in both groups, 8% were taking anti-depressant drugs although, overall, PwE had more severe depressive symptoms, as shown by higher Beck Depression Inventory (BDI-II) scores (9). In a study designed specifically to explore anxiety and depression, Hospital and Anxiety Depression Scale (HADS) scores indicative of anxiety were reported in 50.4% of the subjects, while 39.8 and 46.9%, respectively, had HADS and Patient Health Questionnaire (PHQ-9) scores indicative of depression. In this latter study, female gender and financial problems were significantly associated both with anxiety and with depression. Living in high-income countries decreased the odds for anxiety, while difficulties accessing ASMs increased the odds for depression (12).

One investigation looking specifically at lockdown-related symptoms showed depression in 8.6% and anxiety in 26.7% of subjects (3). In another, 59.4% of the responders reported increased stress (4). In a third survey, 9.6% of PwE and 6.8% of controls reported that since the start of the COVID-19 restrictions, they had begun taking new psychotropic drugs for insomnia (38.2%), depression (14.5%), and anxiety (47.4%) (9). In a subsequent work on the same questionnaire the authors reported on how PWE and PWoE coped with the pandemic restrictions, according to a Natural Language Processing (NLP), examining the single words with which they described how the lockdown changed their life. While words over-reported in the group of PwoE were related to anxiety in the context of a reactive stress response, PWE overexpressed words connected to sadness and worries with their disease. Moreover, PwE expressed positive relief feelings more frequently than PwoE (13).

A further survey evaluated several social and psychological items, asking participants to rate them, on a 10-point Likert scale, for two periods: before vs. after the pandemic emergency. This revealed differences, not large but statistically significant, in the strength of their social support networks, perceived isolation, and levels of anxiety (8).

Finally, behavioral deterioration was reported in 30.3% of a population of children with developmental and epileptic encephalopathies (DEE). Of note, in the same survey, new-onset symptoms of anxiety (68.6%) or depression (69.7%) were reported in caregivers. The main variables associated with behavioral deterioration were type of epilepsy, living in a home without a terrace or yard, and caregiver anxiety (10).

#### Sleep Changes

Sleep changes were reported in 8.2–71.2% (3, 4) and insomnia in 28.2% (3) of PwE. However, in a survey comparing PwE with controls without epilepsy, the quality of sleep did not differ significantly between the two groups: values out of range on the Pittsburgh Sleep Quality Index were reported in 46.9% of PwE and 42.4% of controls. The most affected aspects of sleep were, in decreasing order: subjective evaluation of sleep quality, sleep latency, and sleep duration (9).

#### Lockdown-Related Problems

Postponed neurological tests were reported by 14.5–61% of responders in three studies (3, 9, 12). Issues with drug supply were reported by 2.7–73% (3, 5, 6, 8, 9). Inability to contact their child's neurologist was reported in as many as 62.8% of cases in a Spanish survey aimed at caregivers of children with DEE. (10)

In a large multinational survey, 22.8% of PwE reported financial problems, including difficulty paying housing costs/bills, eating properly and paying for ASMs. These issues were significantly more common in people living in low- to middle-income countries (12).

#### People With Epilepsy's Fears and Worries During the COVID-19 Emergency

Two studies addressed specific fears of PwE during the pandemic. Fears regarding epilepsy in general were reported in 19.6–23.9% of respondents (3, 7). In one of the studies, PwE reported moderate-to-critical worries concerning seizures during the epidemic (24% of cases), lack of professional consultation (41.2%), and medication supply (48.62%) (7). Fear of infection was reported by 14.5% of PwE in the other study (3).

In a further study, participants reported heightened stress due to social reasons (28.2%), fear of SARS-CoV-2 infection (19.2%), and financial reasons (7%) (4).

In a Chinese study, PwE were significantly more concerned about the pandemic than healthy controls, and recorded significantly higher scores on the Kessler Psychological Distress Scale. Furthermore, a higher proportion of PwE had severe distress scores. Higher levels of distress were associated with drug-resistant epilepsy and time spent following the news about COVID-19 (14).

#### Satisfaction With Telemedicine

In two different studies, one settled in Spain (3) and one in Germany (15), 82–83.9% of PwE stated they were satisfied with telemedicine. However, there emerged no clinical predictors of a positive attitude toward it. In the German study, the patients underlined the following advantages of telemedicine: no need for transport (71%), greater convenience (64%), short waiting times (51%), and no travel expenses (41%), while lack of personal contact (44%), and of further diagnostics (45%) were identified as the main disadvantages.

Looking to the future, 38–74% patients saw the usefulness of telephone visits, although the patients in the German study also wanted further appointments onsite, and 36.5% of the Spanish series stated that they preferred face-to-face consultations. In the Spanish study telemedicine was more positively viewed by those most fearful of COVID-19 (3), whereas in the German study a better perception of telemedicine was associated with younger age, not being native German speaker, and a shorter history as a patient at the department. Conversely, longer duration of epilepsy, taking ASMs, and a longer history as a patient at the department were positive predictors of the desire for onsite consultations (15).

#### COVID-19 Confirmed Diagnosis

Several of the reviewed studies collected data on people with alleged symptoms of COVID-19. However, due to the lack of uniformity between these studies, we here focus solely on confirmed diagnoses. The rate of confirmed SARS-CoV-2 infections was 0.2–2.5% (3, 5, 7, 9, 12), which generally corresponded to the expected background for the given country in the same period. In the study by Assenza et al., the rate of infection in people with and without epilepsy was comparable (0.2 vs. 0.4%) (9). One study found no changes in seizure frequency (3), while another reported an increase in three of the nine cases (12).

### Questionnaire

In total, 245 PwE responded to our questionnaire. However, 23 questionnaires were excluded: 12 because the respondent did not consent to data processing; 10 because they appeared to be duplicates (showing the same registration e-mail address and key variables as another response); finally, one questionnaire was excluded as the patient was a minor. In the case of duplicated questionnaires, we kept the most recent. The analyzed questionnaires therefore numbered 222. Responders were identified by consecutive numbers. Their mean age was 43.5 years (range 18–84). Table 2 lists other general characteristics of the population. Most responders (*n*. 201, 90.5%) lived with other people. Thirty-four (15.3%) usually lived in a residential facility or attended a day center; of these, 20 (9%) reported they currently did so. At recruitment, half of the sample (*n*. 114, 51.3%) had been seizure free for 1 year or more. Ninety-nine patients (44.6%) were on ASM monotherapy, 109 (49.1%) on ASM polytherapy, 8 (3.6%) did not use drugs, and 6 (0.9%) did not answer.

**Table 2:** General characteristics of the population.

	**Frequency**	**Percentage**
**Compiler**		
Patient	157	70.72
Parent/caregiver/guardian	65	29.28
*Total*	222	100.00
**Age (categories)**		
18–29	53	23.87
30–39	41	18.47
40–49	46	20.72
50–59	48	21.62
60–84	34	15.32
*Total*	222	100.00
**Sex**		
M	94	42.34
F	128	57.66
*Total*	222	100.00
**Marital status**		
Married	91	40.99
De facto relationship	10	4.50
Divorced/separated	12	5.41
Single	104	46.85
Widowed	5	2.25
*Total*	222	100.00
**Employment**		
Unemployed	35	15.77
Employee	52	23.42
Self-employed	16	7.21
Retired	37	16.67
Student	18	8.11
Other	64	28.83
*Total*	222	100.00
**Education**		
No education/primary school	14	6.31
Secondary school	49	22.07
High school	106	47.75
University degree or higher	46	20.72
*Missing*	7	3.15
*Total*	222	100.00
**Disability**		
No	126	56.76
<100%	42	18.92
100%	52	23.42
*Missing*	2	0.90
*Total*	222	100.00
**Driving license**		
No	101	45.50
Yes	121	54.50
*Total*	222	100.00

Seventy-five patients (33.8%) suffered from additional diseases; 102 (46%) took other medications, besides ASMs. Thirty-one patients (14%) reported a psychiatric diagnosis or current psychiatric therapy; for the analysis, these patients were grouped under the variable “reported psychiatric condition and/or medication.”

Seventy-three patients (32.9%) had at least one seizure after February 23.

One hundred patients (76.6%) reported that their clinical conditions had not changed since the start of the COVD-19 restrictions. Twenty-five patients (11.3%) reported an improvement: great in 11 (5%) and moderate in 14 (6.3%), and 27 (12.2%) a deterioration: moderate in 25 (11.3%) and severe in 2 (1%). However, among those who reported a clinical worsening, 14 (6.3%) had a worsening in seizures, while 13 were seizure free, therefore the worsening did not refer to epilepsy. Among these latter patients, nine had a reported psychiatric condition and/or medication and one had several diseases including cancer. Two patients reported the occurrence of a status epilepticus.

Fifty-three patients (23.9%) reported sleep changes. In particular, 19 (8.5%) reported various degrees or types of disturbed sleep, 25 (11.3%) a change of sleep pattern, and 4 (1.8%) an improvement in sleep.

Thirty persons (13.5%) reported problems with access to healthcare. Among them, seven had problems contacting their general practitioner, and four contacting their treating neurologist.

Eighteen persons (8.1%) reported drug supply problems. They included nine who reported difficulty obtaining Depakin 500 Chrono, which has been in short supply in Italy since the end of March^1^. Three persons had difficulty getting their therapeutic plans renewed (a legal requirement for some medications subject to prescription restrictions in Italy) and seven persons (3.1%) getting their driving license renewed. Forty-two persons (18.9%) stated that they had work/financial problems and 37 (32.7%) had concerns over possible problems linked to the pandemic and related restrictions.

We found statistically significant associations between reported worsening of clinical conditions and disability (20% of persons with disability reported a clinical worsening vs. 6% of those without disability, *X*^2^ = 9.22, *p* = 0.002), reported psychiatric condition and/or medication (42 vs. 7%, Fisher's exact *p* < 0.001), sleep disorders (47 vs. 9%, Fisher's exact *p* < 0.001), changes in social and working life (16 vs. 3%, *X*^2^ = 6.65, *p* = 0.010), and problems with limited access to healthcare (27 vs. 10%, Fisher's exact *p* = 0.016). The multiple correspondence analysis confirmed this pattern of associations, defining in particular a strong association between reported worsening of clinical conditions, a reported psychiatric condition and/or medication and the presence of sleep disorders. We also detected these relationships through univariate logistic regression models, showing a higher probability of reported clinical worsening among individuals with a disability (OR = 3.64, *p* = 0.004, 95% CI = [1.52;8.73]), a reported psychiatric condition and/or medication (OR = 9.13, *p* < 0.001, 95% CI = [3.72; 22.40]), sleep disorders (OR = 9.25, *p* < 0.001, 95% CI = [3.33; 25.71]), changes in their working and social life (OR = 5.69, *p* = 0.021, 95% CI = [1.31; 24.79]), and problems due to limited access to healthcare (OR = 3.31, *p* = 0.012, 95% CI = [1.30; 8.46]). In order to identify statistically significant covariates, controlling for all potential confounding factors, a multivariate logistic regression model was implemented, with a backward elimination criterion. The factors found to increase the probability of reported clinical worsening were: a reported psychiatric condition and/or medication (OR = 12.59, *p* < 0.001, 95% CI = [4.06; 38.99]), sleep disorders (OR = 8.41, *p* = 0.001, 95% CI = [2.31; 30.70]), problems with limited access healthcare (OR = 4.71, *p* = 0.016, 95% CI = [1.34; 16.56]), and experiencing at least one seizure after February 23, as compared with patients reporting seizure freedom lasting 1 year or more (OR = 4.51, *p* = 0.007, 95% CI = [1.51; 13.47]).

Eighty-five persons (38.3%) were opposed to the idea of replacing face-to-face appointments with telephone consultations, while 63 (28.4%) felt that the latter might be useful only occasionally and for minor problems; 53 (23.9%) would accept telephone consultations all or most of the time, while 21 (9.5%) “did not know.” Patients with disability and those who were not seizure free were the least inclined to see telephone consultations or video calls replacing face-to-face contact. In this regard, statistically significant associations were found with disability (*X*^2^ = 12.79, *p* = 0.005), time of last seizure (*X*^2^ = 21.18, *p* = 0.002), and changes in social and working life (*X*^2^ = 12.67, *p* = 0.005).

Two patients (0.9%) self-reported a SARS-CoV-2 infection; one was asymptomatic and the other severe, requiring hospitalization. The latter reported a worsening of epilepsy and in particular “tremor and slightly more frequent seizures.”

## Discussion

The COVID-19 emergency has presented numerous challenges to our way of life, changing routines and generating unprecedented fears and worries. Moreover, it has seriously limited access to healthcare for people with chronic conditions, including epilepsy.

Several groups of researchers, in different parts of the world, have explored the impact of the current emergency on PwE, and our own survey adds to this body of knowledge. We investigated the issues faced by adult PwE during the COVID-19 emergency in Italy, studying a sample probably made up exclusively of patients of a tertiary center that, being based in a hospital temporarily transformed into a COVID-19 hospital, had to reorganize its activities during the pandemic emergency.

Our population mainly comprised patients with moderate to severe forms of epilepsy: almost half of them had experienced seizures during the previous year and almost half were on polytherapy. Moreover, it is a population with a high rate of comorbidities: half of the patients have a disability, 15.3% usually lived in a facility or attended a day center, one third reported additional diseases, and almost half took additional medications.

Our literature review showed that seizure worsening rates varied between populations; however, the majority of people surveyed reported unchanged conditions. In most populations seizures worsened in <10% of PwE (3, 4, 6, 7), a finding that may also reflect the natural fluctuations of epilepsy itself, given that no causal relationship could be established, particularly in studies performed after only 1 month of confinement. In our survey, which was conducted some months after the beginning of the emergency, and thus covered a longer observation period, 6.3% of patients reported seizure worsening, which was among the lowest reported rates. In general, the large majority of patients (76.6%) reported unchanged clinical conditions. The remaining patients were equally distributed between improvement and worsening of clinical conditions, where worsening did not necessarily refer to seizures. Indeed, reported worsening was also significantly associated with disability, reported psychiatric condition and/or medication and sleep disorders. These findings support the view that quality of life in people with epilepsy is multifactorial (16), and are also in line with the results of a survey in children with DEE, in whom behavioral worsening emerged as a major issue (10).

Moderate to high rates of comorbidity with mood disorders, as well as new onset of anxiety, depressive symptoms and heightened stress were reported in the literature (3, 4, 9), and in one study a considerable proportion of individuals started taking psychotropic drugs during lockdown (9). In our population, 14.3% of patients had a reported psychiatric condition and/or medication, which is a relatively low prevalence. However, our questionnaire did not directly address this aspect, nor we did administer any specific scale to assess it. Of note, however, in our population, as well as in others (3, 9), the presence of a reported psychiatric condition and/or medication was one of the main factors associated with reported clinical worsening.

Sleep changes during confinement, including a rise in sleep disturbances, were relatively common in our and other populations (3, 4, 9). However, this is not a specific feature of patients with epilepsy as demonstrated by a survey including controls (9) and by studies in general populations (17). Nevertheless, sleep changes and sleep disturbances were significantly associated with reported clinical worsening both in our and other surveys (3, 9).

The fact that specific emergency-related problems were the ones showing the largest variations between the different populations probably reflects differences in national health system organization, lockdown rules, average incomes and differences in the timing of data collection. In our population, a minority of patients (13.5%) reported problems with limited access to healthcare, the most common being difficulty contacting their general practitioner, which is not surprising given the overwhelming burden placed on GPs during the emergency, and the large number of them (in Italy) who contracted COVID-19. It should be highlighted, however, that problems accessing healthcare were associated with reported clinical worsening both in our population and in others (9, 10).

The low number of people experiencing problems obtaining prescription drugs or getting therapeutic plans and driving licenses renewed possibly reflects the efficiency of the action taken by the Italian government in this regard, namely to postpone legal deadlines until the end of the emergency. Among the 8.1% who reported drug supply problems, half referred to difficulty obtaining Depakin 500 Chrono, which has been in short supply in Italy since the end of March, due to production problems^1^. The observation that the system held up well, compared with other reported data (3, 5, 6, 8, 10), could be attributed at least in part to the fact that, in many Italian regions, ASMs are normally supplied directly by hospitals in large quantities at a time, thereby limiting the risk of shortages for patients.

The decision to resort to telemedicine was favorably viewed by just over half of our patients and around half of these also felt that in future it should be used only on certain occasions and to deal with minor problems. Similarly, despite a very high level of satisfaction with telephone consultations, more than one third of the patients in a Spanish study stated that they would prefer face-to-face appointments in the future (3), while the majority of the patients in a German study agreed to future telemedicine consultations, but only if combined with onsite visits (15). It should be mentioned that for the patients in our study, telemedicine took the form of telephone consultations as we were not equipped for videocalls, and this could have influenced their answers. Moreover, as elsewhere, telemedicine did not allow further diagnostic (15, 18, 19). According to an online survey endorsed by the main national neurophysiological scientific societies, the number of EEGs performed in neurophysiological centers all over Italy dropped by 76% during the 1st weeks of lockdown (18). The COVID emergency has accelerated the implementation of telemedicine in Italy leading also to the issue of specific national recommendations on its use in the context of neurophysiology, including remote inter-hospital consultations (19), which could allow EEG recording while limiting people travels to reach a tertiary center, possibly impacting both on telemedicine effectiveness and on patients' satisfaction.

Neither our survey nor the other reported surveys were designed to provide information on SARS-CoV-2 infection in PwE, however their collective data seem to indicate prevalence rates reflecting those in the general population (9). A worsening of seizures has been signaled in some cases (12), including one of the two patients who reported the infection in our survey.

Our study has several limitations. First, since it concerned a web-based survey, precise information on type of epilepsy was unavailable, and other important clinical information, such as comorbid conditions, was self-reported, and, besides, we did not administer psychometric scales. Second, for the same reason, there was probably a selection bias toward a younger age group and a higher level of education. Third, due to its cross-sectional design, caution is needed when inferring causal relationships. Fourth, respondents were not asked to specify their region of residence, which might have been an important aspect, as different regions in Italy were differently hit by the pandemic. However, a previous Italian report did not find regional differences in any aspect investigated (9).

In conclusion, our survey, in line with others conducted elsewhere, showed that most PwE did not experience a significant change in their clinical conditions as a consequence of home confinement and healthcare reorganization during the COVID-19 emergency. However, severity of epilepsy, concomitant disability, comorbid depression and anxiety, new-onset sleep changes, and limited access to healthcare may affect seizure frequency and other health determinants. Follow-up studies are needed to confirm these observations.

## Data Availability Statement

The raw data supporting the conclusions of this article will be made available by the authors, without undue reservation.

## Ethics Statement

Ethical review and approval was not required for the study on human participants in accordance with the local legislation and institutional requirements. The patients/participants provided their written informed consent to participate in this study.

## Author Contributions

BM and LL conceptualized and designed the study, and drafted the questionnaire and manuscript. CC, LD, LF, VM, CS, PA, FP, and LM collected and interpreted the data. SM performed statistical analysis. FB, PT, and LV critically revised the manuscript for important intellectual content. All authors contributed to the article and approved the submitted version.

## Conflict of Interest

The authors declare that the research was conducted in the absence of any commercial or financial relationships that could be construed as a potential conflict of interest.

## Footnotes

^1^Available online at: https://www.lice.it/LICE_ita/commissione_farmaco/commissione_farmaco.php (accessed September 30, 2020).
