# Supplementary material for: The Impact of the COVID-19 Pandemic on People With Epilepsy. An Italian Survey and a Global Perspective
Source: Front Neurol. 2020 Dec 18;11:613719. doi: 10.3389/fneur.2020.613719 (PMC7775598; doi:10.3389/fneur.2020.613719)
Supplement: Supplementary file 1 [file Data_Sheet_1.PDF]

COVID-19 epidemiological emergency
Impact of containment measures on epilepsy

DATA COLLECTION SHEET

E-mail address* ____

To whom it may concern,
Thank you, in advance, for taking time to fill in this questionnaire, which is designed to assess the direct and indirect effects of the COVID-19 epidemic on patients with epilepsy. In particular, we would like to investigate the impact of COVID-19 infection and of the lockdown measures on patients' lives and the possible consequences on seizure control and lifestyle.

I agree to the use of the data resulting from this questionnaire for research and scientific publication purposes. We inform you that the data you provide when filling out the "Google Forms" questionnaire will be processed in compliance with the provisions in force pursuant to Legislative Decree no. 196/2003, as updated by Legislative Decree no. 101/2018, and GDPR 2016/679. If you fail to agree you will be unable to access the questionnaire. The data obtained from the questionnaire will be used for research and scientific publication purposes as aggregate data, from which it will not be possible to trace your personal identity.*

[1] I agree [2] I don't agree

GENERAL INFORMATION

Questionnaire completed by* [1] Patient [2] Parent/caregiver/guardian (all data entered subsequently must refer to the patient)

Date of compilation* ___/___/___ (dd/mm/yyyy)

PATIENT INFORMATION

Date of birth* ___/___/___ (dd/mm/yyyy)

Age (years)* _______

Sex* [1] M [2] F

Marital status*
[1] Single
[2] Married
[3] Divorced/separated
[4] Widowed
[5] De facto relationship

Employment*
[1] Employee
[2] Self-employed
[3] Student
[4] Unemployed
[5] Retired
[6] Other:_________________________

Education* ____

Disability* | 1 Yes | 2 No

If you answered yes to the previous question, please specify the % of disability ______%

Driving licence* | 1 Yes | 2 No

HOUSING SITUATION

Do you live with anybody? * | 1 Yes | 2 No

If you answered yes to the previous question, please specify who you live with: ____

Do you usually live in a facility/frequent a day center?* | 1 Yes | 2 No

Are you currently living in a facility/frequenting a day center?* | 1 Yes | 2 No

Have you had any problems because of the closure of the center?* | 1 Yes | 2 No

If you answered yes to the previous question, please specify what problems you have had: ____

COVID-19 INFECTION

Have you got COVID-19 infection?* | 1 Yes | 2 No

When did you contract COVID-19 infection?* ___/___/___ (dd/mm/yyyy)

What symptoms have you shown?*

1 Asymptomatic
2 Mild symptoms
3 Moderate symptoms/home care
4 Severe symptoms/hospitalization

Has COVID-19 infection affected your epilepsy?* | 1 Yes | 2 No

If so, please specify how: ____

EPIDEMIOLOGICAL EMERGENCY

Regardless of whether or not you have contracted COVID-19 infection, your clinical conditions since 23/02/2020 (first Legislative Decree on the COVID-19 epidemiological emergency) have: *

1 severely worsened
2 moderately worsened
3 not changed
4 moderately improved
5 greatly improved

If your clinical conditions have changed, please specify in what regard*

1 Seizure frequency
2 Seizure intensity
3 Convulsive seizures
4 Seizures with falls
5 Status epilepticus

	6 Seizure free

CLINICAL INFORMATION

Approximate date of your last seizure*	___/___/___ (dd/mm/yyyy)
Any additional comments on your last seizure:	______________________________
Current therapy*	______________________________
Has your therapy remained unchanged?*	1 Yes 2 No
Do you suffer from any other disease?*	1 Yes 2 No
If you suffer from other disease(s), please specify what:	______________________________
Do you take any other medications besides those for epilepsy?*	1 Yes 2 No
If you take any other medications, please specify them:	______________________________

POSSIBLE CHANGES

Since the beginning of the infection containment measures (approximately since 23/02/2020), have there been any changes in the regularity of your therapy intake?*	1 Yes 2 No
If you answered yes to the previous question, please specify what changes:	______________________________
Since the beginning of the infection containment measures (approximately since 23/02/2020), have there been any changes in your sleep habits?*	1 Yes 2 No
If you answered yes to the previous question, please specify what changes:	______________________________
Since the beginning of the infection containment measures (approximately since 23/02/2020), have there been any changes in your diet?*	1 Yes 2 No
If you answered yes to the previous question, please specify what changes:	______________________________
Since the beginning of the infection containment measures (approximately since 23/02/2020) have there been changes to any aspects of your social and working life?*	1 Yes 2 No
If you answered yes to the previous question, please specify the areas in which you have experienced the biggest changes:	1 Work<br>2 Social interactions<br>3 Mood<br>4 Other:______________________
Have you encountered any problems with limited access to healthcare?*	1 Yes 2 No

If you answered yes to the previous question, please specify:

- [1] Problems accessing the emergency room
- [2] Problems contacting your treating neurologist
- [3] Problems contacting your general practitioner
- [4] Other:________________________________

Have you encountered any problems with medication supply?* [1] Yes [2] No

If you answered yes to the previous question, please specify what problems: ________________________________

Have you encountered any problems over renewal of your treatment plan?*

- [1] Yes
- [2] No
- [3] NA

If you answered yes to the previous question, please specify what problems: ________________________________

Have you encountered any problems over renewal of your driving license?*

- [1] Yes
- [2] No
- [3] NA

If you answered yes to the previous question, please specify what problems: ________________________________

Have you encountered any problems related to work/financial issues?* [1] Yes [2] No

If you answered yes to the previous question, please specify what problems: ________________________________

If you answered no to the previous questions, have you had any other concerns relating to the topics covered? [1] Yes [2] No

If you answered yes to the previous question, please specify what concerns: ________________________________

REMOTE CONSULTING

Would you consider it useful or feasible to replace the classic check-up with a telephone consultation or a video call even after the end of the emergency?*

- [1] Yes, always or in most cases
- [2] Yes, but only occasionally for minor problems
- [3] No, because direct evaluation and the possibility of a face-to-face consultation are necessary
- [4] I don't know

Thank you for answering this questionnaire!

Please write here any remarks you would like to add:

____

*required fields
